# Arctigenin Inhibits Glioblastoma Proliferation through the AKT/mTOR Pathway and Induces Autophagy

**DOI:** 10.1155/2020/3542613

**Published:** 2020-09-15

**Authors:** Yong'an Jiang, Jiayu Liu, Wangwang Hong, Xiaowei Fei, Ru'en Liu

**Affiliations:** ^1^Department of Neurosurgery, Jiangxi Provincial People's Hospital Affiliated to Nanchang University, Nanchang, Jiangxi 330006, China; ^2^Department of Neurosurgery, Peking University People's Hospital, 11th Xizhimen, South St. Beijing 100044, China; ^3^Department of Physiology, Dalian Medical University, Dalian 116044, China

## Abstract

**Purpose:**

Arctigenin (ARG) is a natural lignan compound extracted from Arctium lappa and has displayed anticancer function and therapeutic effect in a variety of cancers. Arctigenin is mainly from Arctium lappa extract. It has been shown to induce autophagy in various cancers. However, as for whether arctigenin induces autophagy in gliomas or not, the specific mechanism is still worth exploring.

**Methods:**

Using CCK8, the monoclonal experiment was made to detect the proliferation ability. The scratch experiment and the transwell experiment were applied to the migration and invasion ability. PI/RNase and FITC-conjugated anti-annexin V were used to detect the cell cycle and apoptosis. Western blotting was used to determine the specified protein level, and constructed LC3B-GFP plasmid was used for analysis of autophagy.

**Results:**

Our research showed that ARG inhibited the growth and proliferation and invasion and migration of glioma cells in a dose-dependent manner (U87MG and T98G) and arrested the cell cycle and induced apoptosis. Interestingly, ARG induced autophagy in a dose-dependent manner. We applied Western blotting to measure the increase in the key autophagy protein LC3B, as well as some other autophagy-related proteins (increase in Beclin-1 and decrease in P62). In order to further explore the mechanism that ARG passed initiating autophagy to inhibit cell growth, we further found by Western blotting that AKT and mTOR phosphorylation proteins (P-AKT, P-mTOR) were reduced after ARG treatment, and we used AKT agonists to rescue, and the phosphorylated proteins of AKT and mTOR increased, and we found that the autophagy-related proteins were also reversed. And interestingly, the protein of apoptosis was also reversed along with autophagy.

**Conclusions:**

We thought ARG inhibited the proliferation of glioma cells by inducing autophagy and apoptosis through the AKT/mTOR pathway.

## 1. Introduction

Glioma is the most common central nervous system tumor, and about 50% is glioblastoma [1]. Surgery, radiation therapy, and drug therapy (temozolomide) are routine ways to treat glioblastoma [2], while the therapeutic efficacy of this disease is not optimistic, and the overall survival time is about 12-18 months. Therefore, there is an urgent need to discover new targets or novel drugs for the treatment of glioma.

The global strategy for the treatment of gliomas has gradually focused on finding new target drugs, and natural biomolecules derived from plants and animal sources are a very rich and available source. Arctigenin (ARG) is a bioactive lignan extracted from Arctium lappa [3] and has various biological activities such as anticancer [4], neuroprotection [5], antioxidation effects [6], antiproliferation [7], and antiviral [8], and ARG exerted a certain therapeutic effect on the treatment of colorectal cancer [9], liver cancer [10], retinoblastoma [11], and prostate cancer [12], and ARG enhances the sensitivity of cisplatin-resistant colorectal cancer cells by activating autophagy and upregulates the levels of apoptosis proteins caspase-3 and caspase-9, thus triggering autophagy-related proteins (LC3 and P62) [9]. It is also reported that ARG inhibits epithelial-mesenchymal transition of hepatocellular carcinoma by suppressing the GSK3*β*-dependent Wnt/*β*-catenin signaling pathway [13]. Moreover, it has been reported in the literature that ARG inhibits the proliferation of retinoblastoma Y79 and promotes apoptosis by downregulating JAG-1 [11]. Although previous studies have demonstrated that ARG inhibits glioma cell proliferation and induces cell cycle arrest and apoptosis [14, 15], the target and mechanism of ARG for the treatment of glioma are still not deep enough, and we need to use further experiments to explore so that we can provide new options for the treatment of glioma.

Previous studies have proved the important role of autophagy in tumorigenesis and treatment [16]. Autophagy is the process of transporting damaged [17], denatured, or aged proteins and organelles to lysosomes for digestion and degradation, which circulates and degrades intracellular components in response to a lack of nutrients or growth factors to maintain homeostasis [18]. Therefore, autophagy is important for maintaining genomic stability and overall cell survival. Microtubule-associated protein light chain 3 (LC3) is localized and accumulated on autophagosomes, so it is considered to be an important sign of autophagy. LC3B transition from LC3B-I to LC3B-II contributes to the formation of autophagosomes. During the autophagy process, autophagosomes engulf cytoplasmic components, including misfolded proteins and discarded organelles. LC3B-I on the autophagy membrane allows the autophagy cargo to interact with the cargo receptor (P62) and can recruit the cargo that degrades to LC3-II when planning to form autophagosomes. Therefore, the expression levels of LC3B and P62 can reflect the activation of the autophagy system. In the cancer and cancer therapy, LC3B and P62 as autophagy markers have complex biological functions. Liver cancer patients with a high expression of LC3B suggest the upregulation of tumor size and serum AFP [19]. The role of P62 needs to be determined to explain its function related to autophagy activity, and the regulation of P62 may play a carcinogenic or tumor suppressive role in cancer [20]. In recent years, many natural extracts have been reported to affect the progression of malignant tumors through autophagy. For example, as a plant hormone, abscisic acid can induce autophagy in glioma cells through the MAPK/JNK signaling pathway [21], and the lectin of Dioclea violacea induces autophagy and causes the death of glioma cell U87MG [22]. Autophagy can be used as a new target for the treatment of tumors and even gliomas. Therefore, there is an urgent need for the development of drugs targeting the autophagy.

However, the role of ARG in the survival of glioma cells and whether autophagy was induced remained unclear. Here, we studied the potential activity of ARG in the treatment of glioma cells, and we confirmed that autophagy did occur in glioma cells treated with ARG. Finally, we also identified the ARG-induced autophagy pathway, namely, the AKT/mTOR pathway. These results provided new ideas for us to treat glioma patients and develop new therapeutic targets to apply clinically.

## 2. Materials and Methods

### 2.1. Chemicals, Reagents, and Antibodies

ARG was purchased from Tianjin Wanxiang Science and Technology Ltd. (Tianjin, China). ARG was dissolved in dimethyl sulfoxide (DMSO) and diluted to 10 mM with phosphate-buffered solution (PBS) and stored at 4°C. Dulbecco's modified Eagle's medium (DMEM) and fetal bovine serum (FBS) (Cambridge, MA) were purchased from Gibco (Grand Island, USA), and antibodies against AKT, P-AKT, mTOR, P-mTOR, MMP2, MMP9, Bax, cleaved caspase-3, Bad, Bcl-2, cyclin E, CDK2, P62, Beclin-1, LC3B, and *β*-actin were purchased from Abcam, and the AKT activator (SC79) was purchased from Beyotime.

### 2.2. Cell Culture

The glioma cell lines (U87MG, T98G) were purchased from the Chinese Academy of Medical Sciences (Beijing, China) and were cultured in Dulbecco's modified Eagle's medium (DMEM) supplemented with 10% fetal bovine serum (FBS) and incubated in an incubator containing 5% CO_2_ at 37°C.

### 2.3. Cell Viability Assay

2 × 10^3^ cells were uniformly cultured in 96-well plates for 24 h, 48 h, 60 h, and 72 h, respectively, and were treated with different concentrations of ARG. 10 *μ*L of CCK8 was added and continued to be incubated at 37°C for 30 min, and the solution was detected by the microplate reader.

### 2.4. Cell Monoclonal Formation Assay

Approximately, 5,000 cells (U87MG and T98G) were cultured in a 10 cm culture dish, and the cells were treated with ARG at a concentration of 100 *μ*M, 200 *μ*M, and 400 *μ*M, respectively, for 15 days. After the treatment, the cells were washed 2-3 times with PBS, fixed with 4% paraformaldehyde for 15 min, and then fixed with crystal violet, and the number of cells was calculated under the microscope.

### 2.5. Wound Healing Assay

All cell lines were cultured in 6-well plates. The cells grew to 85% confluence, scratched with a new 1 mL pipette tip, and washed twice with PBS. The scraped cells were treated with ARG for 24 h or 48 h. ImageJ software was used to capture the image and quantify the gap distance.

### 2.6. Invasion Assay

Transwell was coated with Matrigel (diluted at 1 : 8 with the serum-free medium). 700 mL of DMEM and different concentrations of 20% FBS and ARG (0, 100, 200, or 400 *μ*M) were incubated to the lower transwell compartment. U87MG and T98G were added to the upper compartment at a density of 1 × 10^5^/*μ*L (200 *μ*L/well). After incubating the cells for 24 hours, the remaining Matrigel above the chamber was wiped with a cotton swab. The chamber was fixed with paraformaldehyde and stained with crystal violet for at least 30 min, and four different fields of vision were randomly selected for cell counting to average.

### 2.7. Cell Apoptosis Analysis

The mixture was thoroughly mixed in 500 *μ*L of binding buffer, 5 *μ*L of propidium iodide (PI), and 5 *μ*L of FITC-conjugated anti-annexin V antibody, and the results were measured by the Accuri C6 flow cytometer (BD, USA) within one hour.

### 2.8. Cell Cycle Analysis

1 × 10^5^ cells (U87, T98) were incubated overnight in 6-well plates, and after synchronization with the serum-free medium for 12 h, the cells were treated with different concentrations of ARG. The cells were collected, washed with PBS for 2-3 times, resuspended in 70% ethanol for 24 h, and then washed with ethanol in PBS and added to the mixture with the PI/RNase staining buffer. Finally, the Accuri C6 flow cytometer (BD, USA) was used to measure the results.

### 2.9. Western Blotting

Cell lysates of glioma cells (U87, T98) were used, and proteins were extracted using PRO-PREP™ protein extract (Korea iNtRON Biotechnology). Proteins were separated at different concentrations of sodium dodecyl sulfate-polyacrylamide gel electrophoresis (SDS-PAGE) and transferred to polyvinylidene difluoride (PVDF) membranes (Merck KGaA, Darmstadt, Germany). The membrane was blocked with 5% bovine serum albumin (BSA) for 1.5 h at room temperature and incubated overnight at 4°C with the diluted primary antibody. After incubating the secondary antibody conjugated with HRP for 1 h at room temperature, the results were obtained with electrochemiluminescence (ECL) (Pierce, Rockford, IL, USA).

### 2.10. LC3-GFP Dot Assay

LC3-GFP plasmids were purchased from Addgene; the glioma cells (U87MG and T98G) were transfected with LC3-GFP plasmids and treated with ARG as demanded by experiments and imaged with the fluorescence microscope, and the LC3B-GFP dots after ARG treatment were considered to be autophagic.

### 2.11. Statistical Analysis

The experiment was repeated at least three times, and an independent *t*-test was used to make comparison between the two groups. One-way analysis of variance was used to make comparative analysis among multiple groups on SPSS 20.0 software. *P* < 0.05 was considered statistically significant.

## 3. Results

### 3.1. ARG Inhibited the Growth of Glioblastoma Cells

**Figure 1:**
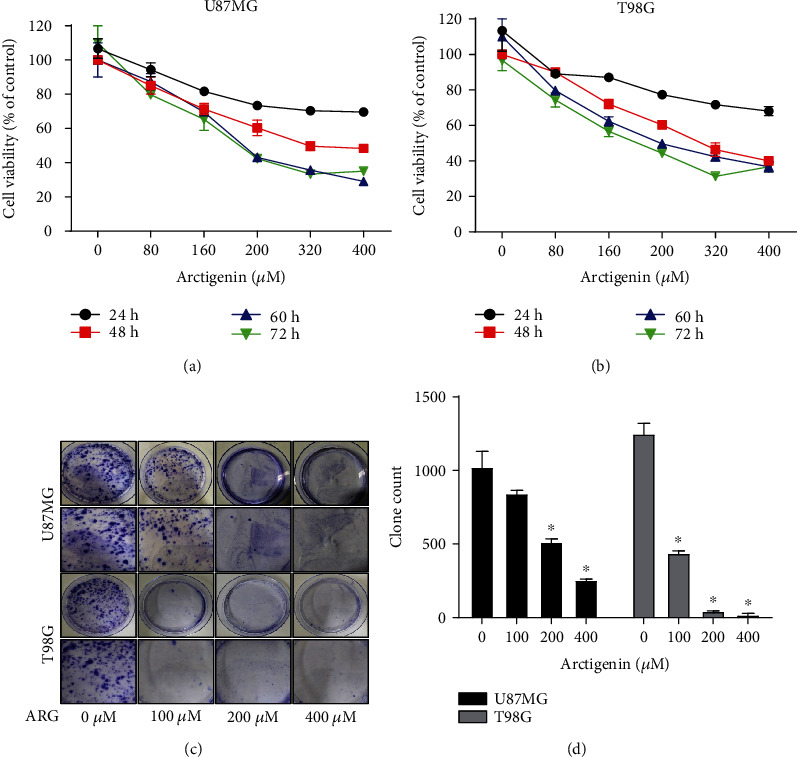
Effect of arctigenin on proliferation in U87MG and T98G. (a, b) The cells were seeded in 96-well plates and were treated with indicated concentrations of ARG for hours by the CCK8 assay. (c, d) 5,000 cells were incubated with 100 *μ*M, 200 *μ*M, and 400 *μ*M ARG for 15 days and were fixed with 4% paraformaldehyde for 15 min and stained with crystal violet for 1 day. Data was expressed as the mean ± standard deviation. ^∗^*P* < 0.05 vs. the control group. All experiments were repeated three times.

To investigate whether ARG affected glioma cell growth or not, we applied the CCK8 assay to detect the cell viability of U87MG and T98G. These glioma cells were treated with different concentrations of ARG in U87MG and T98G, and the cell activity decreased from 60% to 40% when the ARG concentration was increased from 200 *μ*M to 400 *μ*M (Figures 1(a) and 1(b)). We used 100 *μ*M, 200 *μ*M, and 400 *μ*M for the following experiments, and we used the monoclonal formation assay to verify the anchorage-independent growth of glioblastoma cells. After being incubated for 15 days and fixed with crystal violet, ARG-treated glioma cells (U87MG and T98G) were significantly lower than the control group and were concentration-dependent and statistically significant (Figures 1(c) and 1(d)). The results showed that ARG inhibited the cloning of glioma cells.

### 3.2. ARG Inhibited the Migration and Invasion of Glioma Cells (U87MG and T98G)

**Figure 2:**
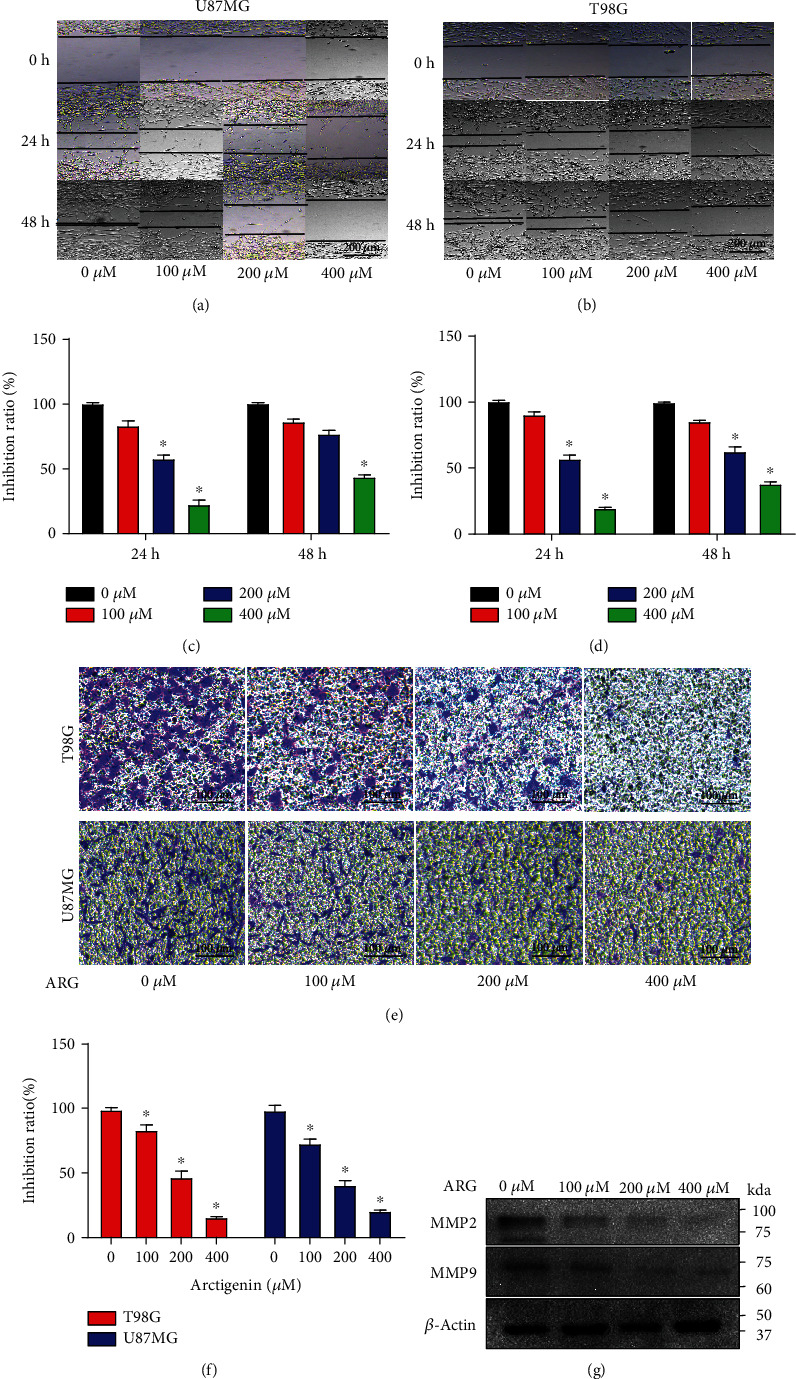
ARG inhibited the migration and invasion of U87MG and T98G in a dose-dependent manner. (a, b) The cells (U87MG and T98G) were incubated in a standard 6-well plate for scratch experiments and were treated with different concentrations of ARG, and pictures were taken under the microscope at 24 h and 48 h. (c, d) Statistical analysis of scratches. (e) U87MG was incubated in the upper chamber of transwell, and different concentrations of ARG (100 *μ*M, 200 *μ*M, and 400 *μ*M) were mixed in the lower chamber of transwell. These cells were stained with crystal violet for at least 30 min, and images were captured with a microscope. (f) Randomly counting the number of cells in the chamber and performing statistical analysis. (g) U87MG was treated with multiple concentrations of ARG for 48 h, and the protein levels of MMP2 and MMP9 were detected by Western blotting, and *β*-actin was used as a housekeeping protein. Data was expressed as the mean ± standard deviation. ^∗^*P* < 0.05 vs. the control group. All experiments were repeated three times.

Previous research studies have revealed that ARG inhibited motility in multiple cancer cells [23]. To determine the effect of ARG on the migration and invasion of glioma cells, the wound healing assay was used to prove the migration ability of U87MG and T98G cells induced by ARG. The cells were incubated by 100 *μ*M, 200 *μ*M, and 400 *μ*M ARG, and then the images of the cells were acquired under the microscope at 24 h and 48 h (Figures 2(a)–2(d)). ARG significantly inhibited the migration of U87MG and T98G in a dose-dependent manner, but not for ARG-treated cells (200 *μ*M) at 48 h. To verify the cell invasion ability, different concentrations of ARG were, respectively, added to incubate the cells (U87MG and T98G) for 24 h. The cells were photographed under the microscope with crystal violet staining. The results indicated that ARG inhibited the invasion of U87MG and T98G in a dose-dependent manner (Figures 2(e) and 2(f)). The expression levels of MMP2 and MMP9 were considered to be an indicator of cell migration and invasion. In our study, the expression levels of MMP2 and MMP9 decreased in ARG-treated cells in a dose-dependent manner (Figure 2(g)). These results indicated that ARG inhibited the invasion and migration ability of glioma cells in a dose-independent manner.

### 3.3. ARG Significantly Inhibited Cell Cycle and Regulated Apoptosis of U87MG and T87G

**Figure 3:**
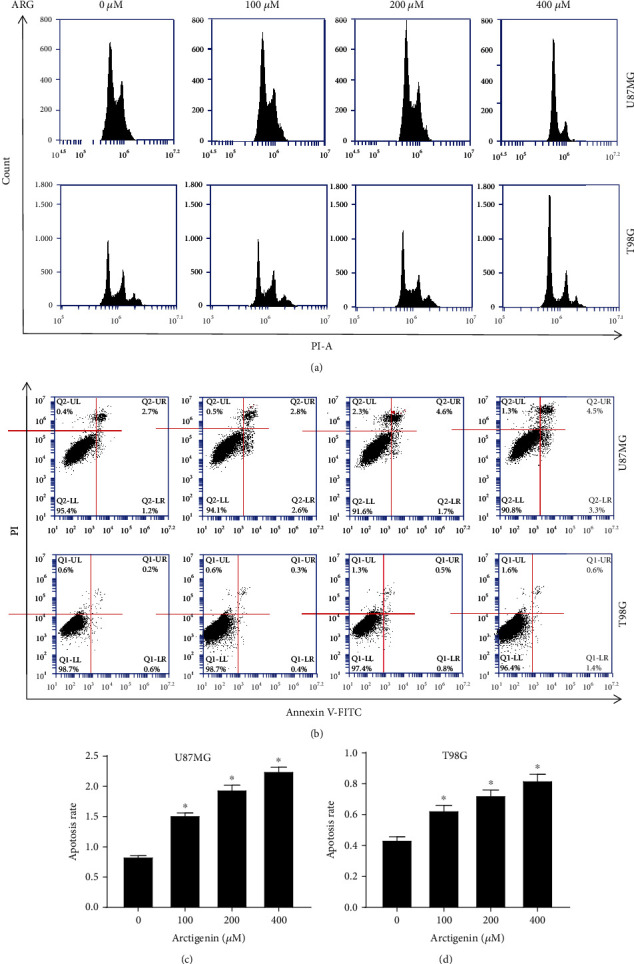
ARG regulated cell cycle and apoptosis of U87MG and T98G. (a) The cell cycle was determined by the PI/RNase staining buffer assay, and U87MG and T98G were, respectively, treated with different concentrations of ARG for 48 h, and then the cells were harvested. The cells were mixed with ethanol overnight, and the results were measured with an Accuri C6 flow cytometry after 15 min. (b) Apoptosis was detected by the PI-FITC-annexin assay and measured within 1 h, and U87MG and T98G were treated with different concentrations of ARG for 48 h, and the cells were analyzed by flow cytometry. The annexin V-FITC axis represented the cells that were early apoptotic, and the PI axis represented the cells in the mid-late stage of apoptosis. Data was expressed as the mean ± standard deviation. ^∗^*P* < 0.05 vs. the control group. All experiments were repeated three times.

Cell cycle and apoptosis were indispensable in exploring the mechanism of antitumor therapy. We used PI/RNase staining buffer to assess the glioma cell cycle progression after treatment with ARG. The toxic effects of ARG on glioma cells (U87MG, T98G) via G1/S phase arrest (Figure 3(a)). Previous articles have shown that cyclin E was detected at the maximum level near the G1/S boundary [24] and that the correlation between the cyclin-dependent kinase 2 (CDK2) and the cell cycle was demonstrated [25]. Western blotting revealed that ARG significantly decreased the level of cyclin E and CDK2 (Figure S1). The results showed that the ratio of G1/S cells of U87MG and T98G treated with ARG increased significantly. To identify whether ARG induced glioma cell apoptosis or not, the effect of ARG on apoptosis of glioma cells (Figures 3(b) and 3(c)) was determined by PI-FITC-annexin. Compared with the control group, apoptosis in cells treated with ARG markedly increased (Figure 3(b)). Since AKT was a key mediator of cell survival by inhibiting proapoptotic proteins (including Bad and Bax) [26] as revealed in additional file 1, the protein expression level of Bad and Bax increased while the protein expression of Bcl-2 and another apoptotic protein cleaved caspase-3 was markedly reduced along with the increase of ARG in a dose-dependent manner. Therefore, we confirmed that the ARG induced cell cycle G1/S arrest and apoptosis.

### 3.4. ARG Regulated Levels of Autophagy-Associated Proteins in a Dose-Dependent Manner

**Figure 4:**
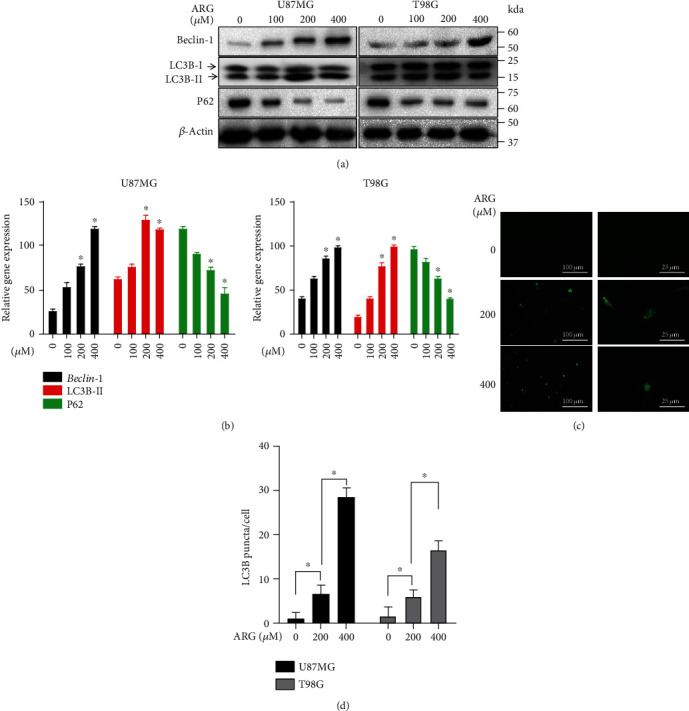
ARG induced autophagy. (a, b) U87MG and T98G were treated with different concentrations of ARG, and the whole cell lysate was subject to LC3B-II and P62 immunoblotting. *β*-Actin was used as a housekeeping protein. (c) U87MG and T98G were transfected with LC3B-GFP plasmids as the control, and the LC3-GFP puncta were observed under the fluorescence microscope. (d) Counting on the number of LC3B-GFP puncta per cell. Data was expressed as the mean ± standard deviation. ^∗^*P* < 0.05 vs. the control group. All experiments were repeated three times.

We speculated whether the biological activity of ARG on U87MG and T98G was mediated by the process of autophagy or not. Therefore, we further used Western blotting to reveal the expression of important autophagy-associated marker factors (LC3B, Beclin-1) and essential-regulated autophagy adapter protein (P62) (Figures 4(c) and 4(d)). We found that the expression level of Beclin-1 and LC3B-I to LC3B-II increased after ARG treatment of cells, while the expression level of P62 decreased. In autophagy induction, LC3B-I was modified and converted to LC3B-II related to fluorophores by combining with lipid phosphatidylethanolamine [27]. Therefore, LC3-II levels were used as an indicator of the number of autophagosomes. The cells were treated at a concentration of 100 *μ*M, 200*μ*M, and 400 *μ*M for 48 h. To further evaluate the role of ARG in inducing autophagy, we established a stable cell line expressing LC3B-GFP to show the formation and occurrence of autophagy (Figures 4(e) and 4(f)). By quantitatively analyzing the number of GFP-LC3 spots, we observed the accumulation of autophagosomes visible in cells transfected by ARG treatment (200 *μ*M, 400 *μ*M) for 48 h. Compared with the control group, we found that U87MG and T98G cells transfected with LC3B-GFP were observed under the fluorescence microscope. The number of autophagosomes increased significantly. Therefore, we believed that one of the reasons for ARG affecting glioma cell proliferation might be due to the AKT/mTOR-mediated autophagy.

### 3.5. ARG Regulated Glioma Cells through the AKT/mTOR Pathway

**Figure 5:**
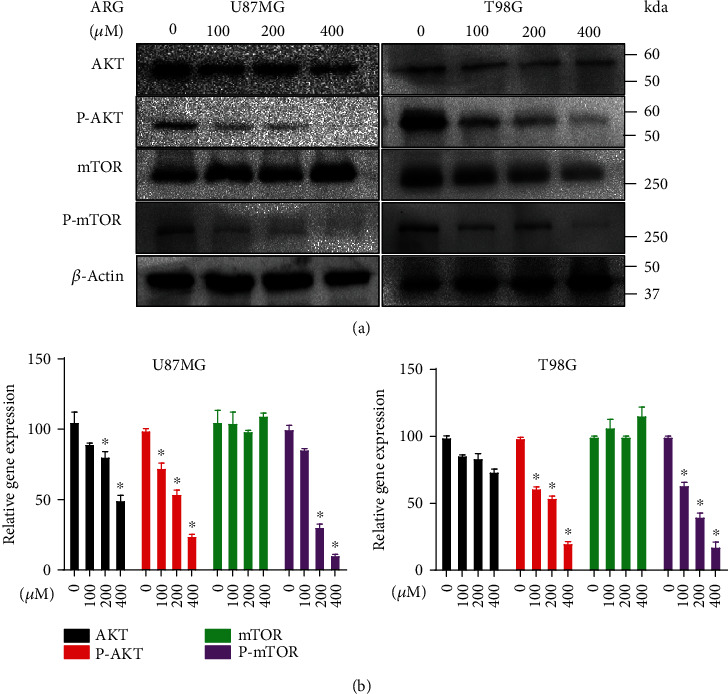
ARG regulated the AKT/mTOR protein pathway. (a) U87MG and T98G glioma cells were treated with different concentrations of ARG for 48 h, and then the protein expression levels of AKT, mTOR, and phosphorylated AKT and phosphorylated mTOR continued to be analyzed; *β*-actin was used as a housekeeping protein. (b) Furthermore, the ImageJ software was used for statistical analysis of the Western blotting results. Data was expressed as the mean ± standard deviation. ^∗^*P* < 0.05 vs. the control group. All experiments were repeated three times.

Many literatures supported that autophagy was mediated by the AKT/mTOR pathway to inhibit tumor growth [28, 29]. In the above, we determined that ARG induced autophagy. Next, we continued to explore possible pathways or mechanisms for ARG-induced autophagy. After treatment with ARG at a concentration of 100 *μ*M, 200 *μ*M, and 400 *μ*M for 48 h, the treated glioma cells (U87MG, T98G) significantly reduced the expression of phosphorylated AKT and mTOR and correspondingly regulated the expression of phosphorylated AKT and mTOR, but there were no significant changes in AKT and mTOR of the total protein level (Figures 5(a) and 5(b)). mTOR regulated the process of autophagy and was one of the main regulators of the process of autophagy. Upstream of it was the PI3K/AKT pathway regulating mTOR activity; therefore, the results indicated that ARG inhibited the growth of glioma by triggering autophagy through the AKT/mTOR pathway.

### 3.6. SC79 Significantly Rescued Autophagy Induced by ARG in Glioma Cells

**Figure 6:**
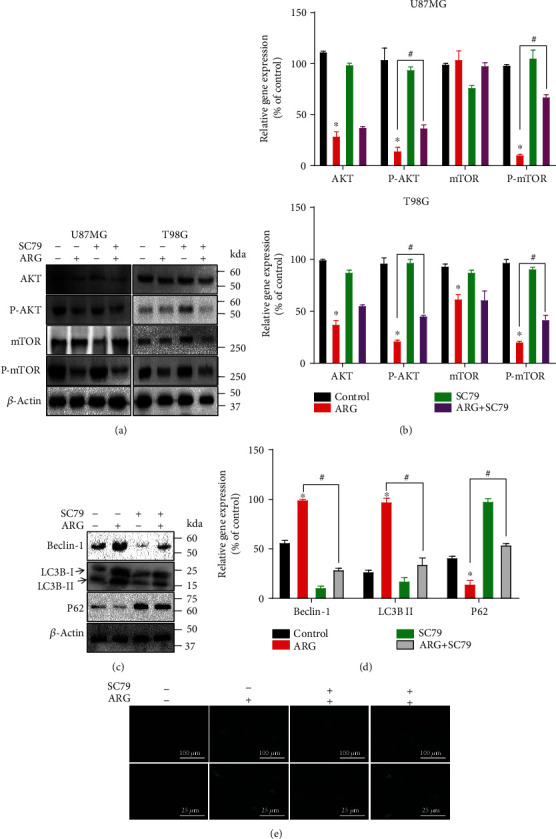
SC79 regulated glioma cell autophagy and apoptosis. (a, b) After 48 h of incubation with ARG and cotreated with SC79 (5 *μ*g/*μ*L), the cells were lysed and used for Western blotting. (c, d) To verify the protein expression levels of AKT, P-AKT, mTOR, and P-mTOR, Western blot was used to ensure the protein level of Beclin-1, LC3B, and P62. *β*-Actin was used as a housekeeping protein. (e) Quantification of the number of GFP-LC3 puncta per cell. Data was expressed as the mean ± standard deviation. ^∗^*P* < 0.05 vs. the control group. All experiments were repeated three times.

Previous experiments have determined that ARG inhibited glioma cells through the AKT/mTOR-mediated autophagy. It was previously demonstrated that ARG might induce autophagy by AKT/mTOR, and to further confirm this, we used an agonist, SC79, an activator of AKT [30], to restore the ability of the AKT pathway. We found that glioma cells of U87MG and T98G were incubated in ARG and SC79 for 48 h. After treating U87MG and T98G with ARG, we discovered an increase in the expression of phosphorylated protein of AKT and mTOR after using SC79 (Figures 6(a) and 6(b)). In this way, we were surer that ARG inhibited glioma cell proliferation and growth through the AKT/mTOR pathway. We further used Western blotting assay to verify autophagy-related proteins. Obviously (Figures 6(c) and 6(d)), SC79 reduced Beclin-1 and the conversion of LC3B-II, and P62 protein levels increased (ARG group vs. ARG+SC79 group), and then U87MG and T98G cells transfected with LC3B-GFP were pictured under the fluorescence microscope (Figure 6(e)); LC3B puncta per cell (Figure S2) were performed. We observed that the number of LC3B puncta was significantly increased (ARG group vs. ARG+SC79 group).

## 4. Discussions

As the main source of therapeutic drugs, plant extracts are increasingly showing that they could greatly prolong the survival of patients and are potential medical treasures in the future [31–33]. As an extract of Arctium lappa, arctigenin (ARG) has an important potential for anticancer, and there are reports that it also has therapeutic effects in the treatment of cancer [34]. As the most common primary tumor of the central nervous system, gliomas are increasing year by year, but the prognosis of gliomas is still very poor. The median survival time is only 5 years [35]. More new treatment methods are urgently needed to be found. In this research, we demonstrated that ARG mediated the anticancer properties of autophagy by targeting the AKT/mTOR pathway in glioma cells.

The proliferation of tumor cells is an important biological feature of tumors; therefore, we provided new methods for tumor treatment by suppressing tumors. Our current research determined that ARG inhibited the proliferation and growth of glioma cells (U87MG and T98G) in a dose-dependent manner. In addition, ARG has been shown to inhibit proliferation of human hepatoblastoma cells (HUH-6 cells) [36] and liver cancer tumors (HepG2, Hep3B) [37].

Cell proliferation and cell division were very closely related, and the only study on gliomas showed that ARG blocked the cycle in a concentration-dependent manner, and blocked the cycle in G 0/G1, and reduced cyclin D1 protein levels [14]. We continued to explore the cell cycle in a broad range. We found that glioma cells after ARG treatment regulated the levels of CDK2 and cyclin E protein. They were also blocked in the G1/S phase. In addition, ARG blocked the G2/M1 cell cycle by downregulating cyclin A, cyclin E, and CDK2, thereby inhibiting the growth of colorectal cancer cells [38]. Although ARG had different effects on different tumors, cycle arrest had been proven in various studies deeply.

Tumor metastasis is still a difficult problem that needs to be overcome in the treatment of tumors. Inhibiting the invasion and migration of tumor cells and inhibiting tumor metastasis can greatly help tumor treatment. In our study, it was found that ARG inhibited the invasion and migration of glioma cells (U87MG and T98G) in a dose-dependent manner. In addition, ARG inhibited the metastasis of human breast cancer cells by reducing the activity of MMP2, MMP9, and heparanase protein [23].

Apoptosis is an important way of the cell biological process, and inducing apoptosis has also become the modern way of treating cancers. The study found that ARG induced apoptosis of primary exudative lymphoma cells in the absence of glucose and induced apoptosis of colon cancer cells through the ROS/p38MAPK pathway [39].

Autophagy, as an important mechanism for maintaining cell homeostasis, has always been a hotspot in the discussion of tumor therapy. Whether autophagy is used as a tumor inhibitor or promoter has been debated. ARG activated autophagy to enhance the sensitivity of cisplatin-resistant colorectal cancer cells [9]. ARG also reduced the ER expression in ER-positive human breast cancer cells by inhibiting the activation of autophagy [40]. In order to determine the autophagy of ARG, analysis results of LC3B, P62, and Benlin-1 in Western blotting showed that ARG enhanced autophagy in a dose-dependent manner. We continued to explore pathways that mediated autophagy in the following.

The blocking of the AKT/mTOR signaling pathway played a crucial role in regulating autophagy [41, 42]. Through the mTOR pathway, targeting NLGN3 secretion promoted the proliferation of gliomas [43]. RIOL3 promoted the proliferation and survival of gliomas through the AKT/mTOR signaling pathway [44] In our study, we identified the phosphorylation of AKT and mTOR inactivated in ARG-treated glioma cells. Therefore, we believed that the reason for the inhibition of glioma cell proliferation with ARG might be mediation by AKT/mTOR caused by autophagy. In this study, SC79 was an AKT activator that activated the activity of AKT. In the presence of SC79, the protein levels of P-AKT and P-mTOR were partially restored, including the expression level of the autophagy marker protein LC3B which also dropped. Previous reports have reported that miR-1271, as a tumor suppressor in pancreatic cancer, promoted apoptosis through the reduction of AKT/mTOR signaling. In our study, it was interesting to find that after using SC79 (AKT agonist), the apoptosis caused by ARG was effectively reduced (data not shown), and the results were detected by Western blotting. We found that there was a close relationship between apoptosis and autophagy. Carvedilol inhibited autophagy and promoted apoptosis in hepatic stellate cells, and the late inhibition of autophagy preceded the induction of apoptosis [45], and one research indicated that inhibited apoptosis protease caspase-8 caused cell death and autophagy enhancement, and inhibition of autophagy-related genes saved cell death [46]. Under starvation or other stress conditions, Bcl-2 and Bcl-xL must replace Beclin-1 to initiate autophagy [47]. These previous studies reminded us that the mechanism by which ARG inhibited glioma cells might not only trigger autophagy but also involve the participation of apoptosis molecules or the mutual conversion of apoptosis and autophagy. To this end, further research studies on the mechanism of ARG's role in gliomas were required, and more targets were needed for the treatment of gliomas. In addition, due to the limitations of laboratory conditions, our research did not involve in vivo experiments and failed to study the characteristics of ARG at the level of biological individuals, but the previous research studies also paved the way for future exploration of gliomas. In conclusion, our data indicated that ARG mediated autophagy by targeting the AKT/mTOR pathway and inhibited the malignant behavior of glioma cells.

## 5. Conclusions

To sum up, ARG as a natural living active molecule for cancer treatment was worthy of our further study and provided a new direction for the treatment of gliomas.

## Data Availability

The data used to support the findings of this study is available from the corresponding author upon request.

## Supplementary Materials

Supplementary Materials Additional file 1, Figure S1: (a) glioma cells (U87MG) treated with different concentrations of ARG for 48 h and Western blot of apoptosis protein (Bax, cleaved caspase-3, Bad, and Bcl-2) and cell cycle protein (cyclin E, CDK2). *β*-Actin acted as a housekeeping protein. (b) Statistical analysis of the protein level by the ImageJ software. Data are expressed as the mean ± standard deviation. ^∗^*P* < 0.05 vs. the control group. All experiments were repeated three times. Additional file 2, Figure S2: (a) statistical analysis of LC3B-GFP puncta per cell. Data are expressed as the mean ± standard deviation. ^∗^*P* < 0.05 vs. the control group. All experiments were repeated three times.
