# Supplementary material for: Arctigenin Inhibits Glioblastoma Proliferation through the AKT/mTOR Pathway and Induces Autophagy
Source: Biomed Res Int. 2020 Sep 15;2020:3542613. doi: 10.1155/2020/3542613 (PMC7512051; doi:10.1155/2020/3542613)
Supplement: Supplementary Materials — Additional file 1, Figure S1: (a) glioma cells (U87MG) treated with different concentrations of ARG for 48 h and Western blot of apoptosis protein (Bax, cleaved caspase-3, Bad, and Bcl-2) and cell cycle protein (cyclin E, CDK2). β-Actin acted as a housekeeping protein. (b) Statistical analysis of the protein level by the ImageJ software. Data are expressed as the mean ± standard deviation. ∗P < 0.05 vs. the control group. All experiments were repeated three times. Additional file 2, Figure S2: (a) statistical analysis of LC3B-GFP puncta per cell. Data are expressed as the mean ± standard deviation. ∗P < 0.05 vs. the control group. All experiments were repeated three times. [file 3542613.f1.pdf]

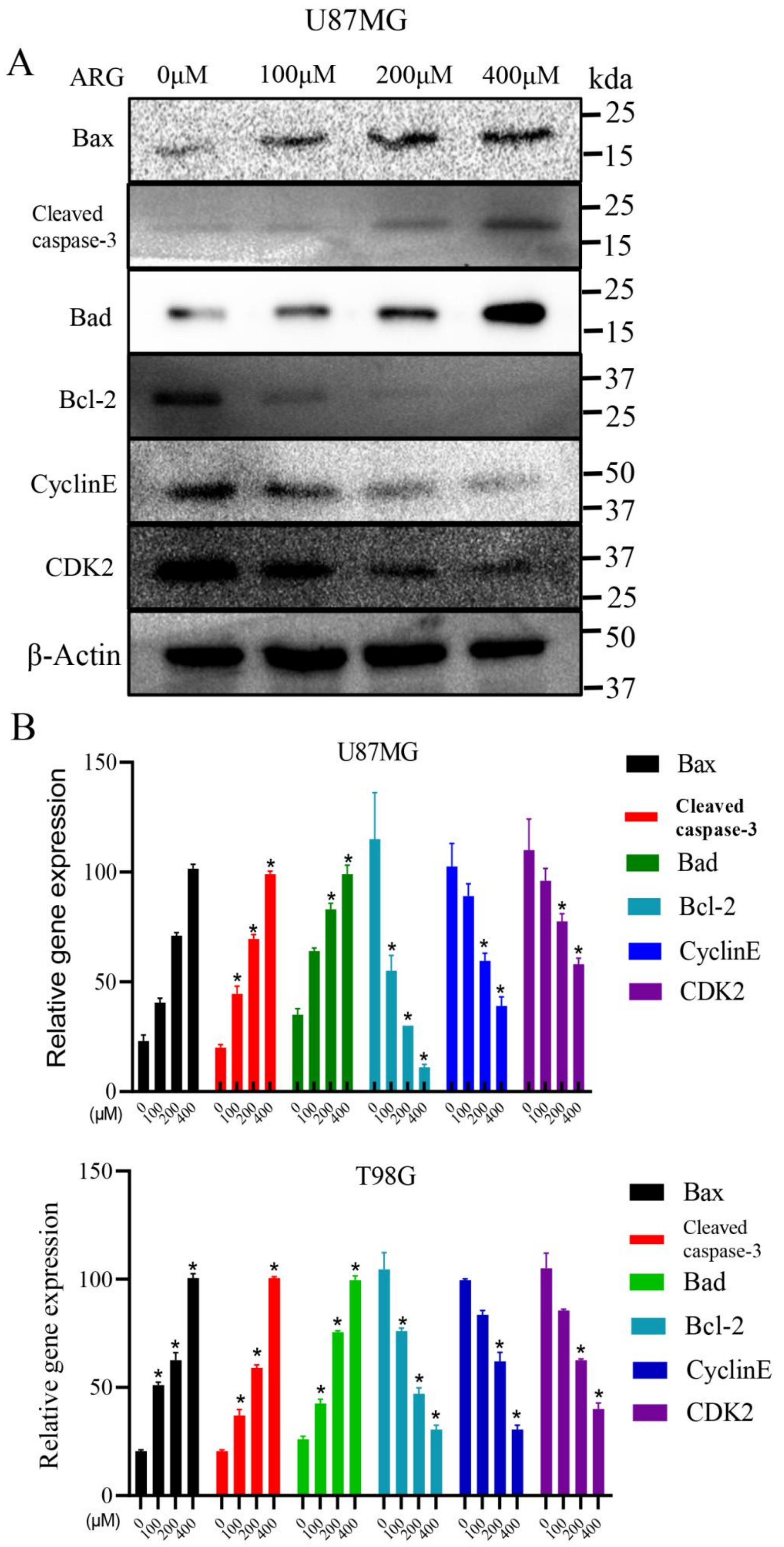
U87MG
A
ARG 0μM 100μM 200μM 400μM kda
Bax
Cleaved caspase-3
Bad
Bcl-2
CyclinE
CDK2
β-Actin
25
15
25
15
25
15
37
25
50
37
37
25
50
37
B
U87MG
Relative gene expression
150
100
50
0
(μM) 0 100 200 400
Bax
Cleaved caspase-3
Bad
Bcl-2
CyclinE
CDK2
T98G
Relative gene expression
150
100
50
0
(μM) 0 100 200 400
Bax
Cleaved caspase-3
Bad
Bcl-2
CyclinE
CDK2

Additional file 1,Figure S1:(**A**)Glioma cells(U87MG) treated with different concentrations ARG for 48h, and western blot of apoptosis protein(Bax,Cleaved caspase-3,Bad,Bcl-2),and cell cycle protein (CyclinE,CDK2). β -actin acted as housekeeping protein.(**B**) statistical analysis of protein level by the image J software. Data are expressed as the mean ± standard deviation.*P<0.05 vs the control group. All experiments were repeated three times.

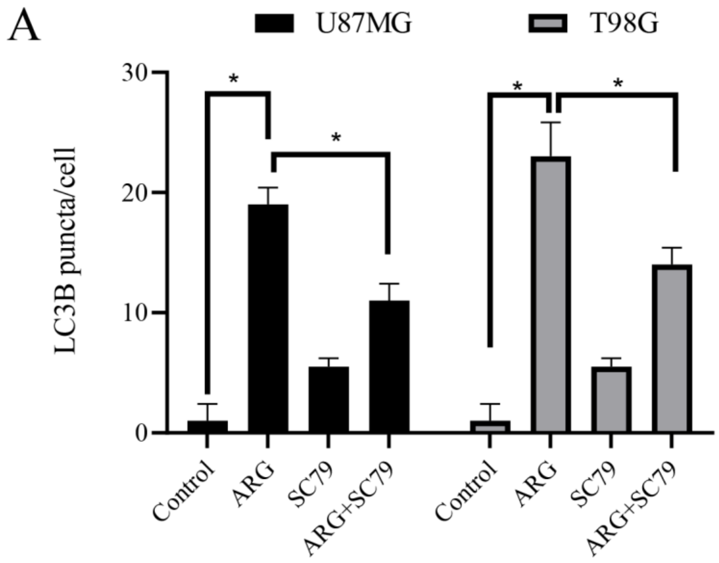

Additional file 2,Figure S2(A) statistical analysis of LC3B-GFP puncta per cell, Data are expressed as the mean ± standard deviation.*P<0.05 vs the control group. All experiments were repeated three times.
